# Impact of Long-Term Home and Community Use of a Lower Limb Sensory Neuroprosthesis

**DOI:** 10.21203/rs.3.rs-7412528/v1

**Published:** 2025-09-19

**Authors:** Aarika Sheehan, Ricardo Siu, Melissa Schmitt, Jillian Vala, John Wright, Daekyoo Kim, Suzhou Li, Emily Graczyk, Ronald J Triolo, Hamid Charkhkar

**Affiliations:** 1 Louis Stokes Cleveland Veterans Affairs Medical Center, 10701 East Boulevard, Cleveland, OH, 44106, USA; 2 Department of Biomedical Engineering, Case Western Reserve University, 10900 Euclid Avenue, Cleveland, OH 44106, USA

**Keywords:** neuroprosthesis, sensory restoration, lower-limb amputees, peripheral nerve stimulation, limb loss, long-term, home use, community use, sensation, sensory feedback

## Abstract

**Background:**

Individuals with lower limb loss experience sensorimotor deficits that often lead to adoption of compensatory gait strategies, which increase their risk of developing secondary comorbidities, reduce balance confidence, and negatively impact functional abilities. Despite advancements in prosthetic technology, current devices lack direct sensory feedback that replicates the somatosensation once provided by the missing foot. This study examines how long-term home and community use of a sensory neuroprosthesis (SNP) designed to provide real-time plantar sensory feedback through implanted nerve cuff technology affects functional performance, balance confidence, and quality of life.

**Methods:**

A 68-year-old individual with unilateral transtibial limb loss utilized the system in home and community environments for 9.5-months to perform normal daily activities, while returning to the laboratory periodically to complete various functional and qualitative assessments.

**Results:**

Results included measurable improvement in prosthetic propulsion, adoption of more typical gait behaviors, reduced errors in stair negotiation, and a decline in self-reported stumble frequency.

**Conclusions:**

These findings offer compelling evidence that home use of the SNP may reduce reliance on compensatory gait mechanics, potentially lower fall risk, and improve user confidence in diverse and uncontrolled real-world environments.

**Trial registration:**

ClinicalTrials.gov Identifier: NCT03409133 – Trial Registration Date: 01/24/2018

## Background

Individuals living with lower limb loss face many functional challenges and are likely to experience psychosocial deficits following amputation [1,2]. Although post-amputation rehabilitation and lower limb prosthesis use help restore basic locomotor function, vital sensory feedback once provided by the missing limb is still lacking. This sensory information is critical for coordinating movement, adjusting balance, and safely navigating uneven terrain [3].

In the absence of somatosensation, individuals rely on compensatory mechanisms to maintain stability, which include increased dependance on visual feedback [4], utilization of pressure cues from the prosthetic socket [5], overuse of the intact limb [6], and reduced gait initiation velocity [7]. However, these strategies are often insufficient, as lower limb prosthesis users continue to exhibit reduced balance confidence [8], increased fall rates [9], and elevated ambulatory energy expenditure [10] compared to persons without limb loss. Moreover, many compensatory strategies are postulated to contribute to the development of secondary impairments, such as osteoarthritis and low back pain [11,12]. These debilitating conditions are more prevalent in individuals with lower limb loss than the general population [12,13], and can lead to activity avoidance, reduced social engagement, and diminished quality of life [11,12,14,15]. With the number of Americans living with limb loss projected to reach 3.6 million by the year 2050 [16], it is imperative to develop prosthetic technologies that improve gait symmetry, normalize biomechanics, and reduce the need for such compensatory gait strategies.

Advanced prosthetic devices such as microprocessor controlled hydraulic knees or powered ankles can improve gait biomechanics and reduce energy expenditure associated with ambulation [17–23]. However, no commercially available prosthesis directly communicates to the nervous system such that users can perceive interactions with the environment. Although sensory substitution approaches such as mechanical vibration or electrocutaneous stimulation to the surface of the residual limb offer alternative forms of feedback, they have not shown major improvements in functional outcomes and pose certain challenges to long-term use, as individuals need to learn to interpret responses that are highly variable and affected by electrode placement, skin properties, and level of attention [24–26].

To overcome such limitations of existing lower limb prosthetic options, our team and others have utilized neural interfaces to activate remaining afferent pathways in the residual peripheral nerves to restore meaningful sensation about the missing extremity [27–29]. Findings from these studies suggest that sensations directly related to the location and magnitude of pressure applied to the sole of the prosthetic foot and perceived as arising from the missing limb can be reliably evoked in people with transtibial or transfemoral limb loss [27,30,31]. These electrically elicited plantar sensations affect the perception of prosthesis weight [32], improve balance and gait symmetry [33], enhance performance during ambulatory searching tasks [34], and reduce metabolic cost of ambulation [28]. However, their long-term effects during activities of daily living in uncontrolled environments are largely unknown.

In the upper limb, home use of a SNP improved functional performance, prosthesis embodiment, and quality of life [35]. However, upper limb tasks are dominated by fine motor movement, while lower limb function demands dynamic stability and coordinated control between body parts. Therefore, we propose that prolonged use of a sensory-enabled prosthesis during daily activities at home and in the community will enhance stability, safety, and confidence for lower limb prosthesis users, particularly during tasks that require dynamic and coordinated control, such as stair negotiation.

Here, we report findings from a 9.5-month home use trial of a SNP designed to elicit real-time sensations related to plantar pressure distribution in a transtibial prosthesis user. Functional, subjective, and qualitative assessments were performed at multiple time points to ascertain how long-term, real-world use of the system affected functional mobility, stair negotiation, and quality of life. We hypothesized that the integration of real-time, context-dependent, and anatomically relevant sensory information into daily activities would enhance gait symmetry, improve stair negotiation, and optimize overall functional outcomes, while reducing reliance on compensatory gait strategies and boosting user confidence in independent and spontaneous navigation of complex environments. The resulting observations have the potential to inform the transition of such systems from the laboratory to real-world applications and demonstrate the feasibility of sensory-enabled prostheses for unsupervised long-term use.

## Methods

### Research Participant

A 68-year-old male with unilateral transtibial limb loss volunteered to enroll in this study after completing informed consent procedures approved by the Institutional Review Board of the Louis Stokes Cleveland VA Medical Center and the Navy Human Research Protection Program. Amputation was due to traumatic injury and occurred 24 years prior to initiation of this home use trial. He was a regular prosthesis user and K3 ambulator with no medical history of peripheral neuropathy or uncontrolled diabetes. The trial was conducted 1.5 years after initial receipt of surgically implanted multi-contact stimulating nerve cuff electrodes [27] followed by laboratory-based experiences with the SNP. Prior to initiation of the home use trial, the system recipient attended regular laboratory sessions to receive neural stimulation and complete procedures to determine sensory thresholds, map locations and quality of the elicited percepts, quantify psychometric properties, assess effects on gait and balance, and other experiments as described elsewhere [27,34,36–38]. Sensory percepts for this participant have been reported in Shell et al. 2021 as LL03 [36]. All study procedures were conducted under an Investigational Device Exemption obtained from the United States Food and Drug Administration in accordance with all applicable guidelines and regulations.

### Sensory Neuroprosthesis

**Figure 1.**
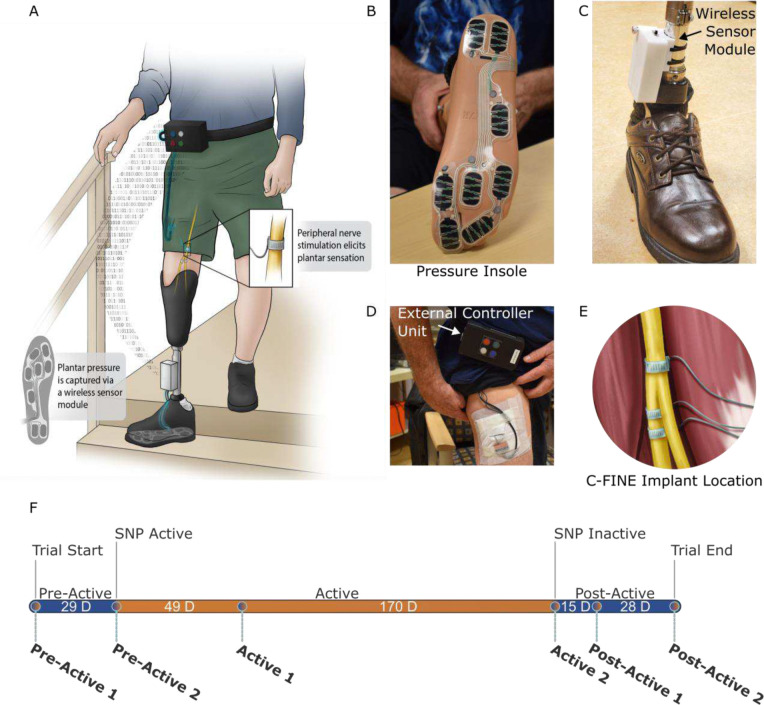
The components of the sensory neuroprosthesis (SNP) for home and community use. Functional communication between the (A) SNP components include a (B) pressure insole, (C) wireless sensor module, (D) external controller unit (ECU), and (E) implanted C-FINEs which are connected to the ECU via percutaneous leads. (F) Timeline of 291-day home use trial consisting of 3 phases (Pre-Active SNP, Active SNP, and Post-Active SNP) and 6 evaluation time points (Pre-Active 1 & 2, Active 1 & 2, and Post-Active 1 & 2). The participant received sensory neural stimulation while performing typical daily activities at home and in the community ONLY during the Active SNP phase. Although the SNP was donned during the Pre- and Post-Active SNP phases, it was inactive and not providing somatosensory feedback.

The SNP was designed to integrate seamlessly with the user’s personal prosthesis, without altering the suspension or socket fit. A pressure sensitive insole (IEE S.A., Luxemburg), secured inside the shoe beneath the prosthetic foot, measured plantar pressure magnitude and locations during walking (Figure 1B). Insole data was digitized by a wireless sensor module (Figure 1C) and transmitted to an external controller unit (ECU, Figure 1D) which dynamically adjusted stimulation parameters in real time.

The ECU delivered charge-balanced, biphasic, cathodic-first pulses via percutaneous leads to selected contacts on two implanted 16-contact Composite Flat Interface Nerve Cuff Electrodes (C-FINEs) implanted around the sciatic and tibial nerves above the knee on the posterior aspect of the residual limb (Figure 1E). Pulse width (PW) ranged from 40–250 μs, pulse amplitude (PA) from 0.3–7 mA, with a fixed interpulse interval (IPI) of 50 milliseconds. Contacts were chosen to evoke sensations that referred to the toes, midfoot, and heel of the missing foot. C-FINE implantation and lead routing were performed in an outpatient surgery as previously described [27,30].

At baseline and at each evaluation time point (as shown in Figure 1F), stimulation parameters were calibrated in the laboratory to ensure comfort and consistency. Initial PA and IPI values were based on prior sensory thresholding experiments and could not be adjusted by the user [27]. Perceived pressure intensity in each foot region was adjusted by modulating PW linearly according to equation 1,

(1) $$PW(t)=\left\{\begin{array}{ll} \frac{PW_{ref}-PW_{min}}{F_{ref}-F_{min}}F(t), & 0\leq F(t)<F_{ref}\\ \frac{PW_{max}-PW_{ref}}{F_{max}-F_{ref}}F(t), & F_{ref}\leq F(t)\leq F_{max} \end{array}\right.$$

where F(t) represents the summed instantaneous plantar force from the insole regions mapped to that foot region. $F_{\min}$, $F_{\mathrm{ref}}$, and $F_{\max}$ correspond to forces measured with the prosthetic foot unloaded, at approximately 50% of body weight, and 100% of body weight, respectively. The associated $PW_{\min}$, $PW_{\mathrm{ref}}$, and $PW_{\max}$ elicited (1) barely perceptible sensation, (2) a sensation matching the pressure of the intact limb when standing with equal weight on both legs, and (3) the maximum comfortable intensity, respectively[39]. To ensure safe stimulation ranges, ${PW}_{\min}$ and ${PW}_{\max}$ were set and adjusted only by study staff. Comfort was confirmed by having the participant walk for at least two minutes in the lab with the SNP system active.

The user could recalibrate $F_{\min}$, $F_{\mathrm{ref}}$, and $F_{\max}$ independently for each foot region. $F_{\min}$ was recorded with the prosthetic foot unloaded; $F_{ref}$ and $F_{max}$ were recorded with approximately 50% and 100% body weight applied to the target region. Midfoot loading was isolated by pressing the midfoot against a step edge. Each position was held for 5 seconds and sensor values averaged. Calibration was accepted only if $F_{\max}>F_{\mathrm{ref}}>F_{\min}$; otherwise, the process was repeated or prior values retained. Upon acceptance, the ECU remapped the PW values to the new force thresholds, which were stored until the next calibration. The user could also fine-tune $PW_{\mathrm{ref}}$ within the preset $PW_{\min}$ and $PW_{\max}$ bounds established in the lab through the ECU interface, effectively shifting the stimulation range to decrease or increase the perceived intensities produced by the SNP.

Prior to home use, the participant received an operating manual and in-laboratory training on the proper use of the ECU. The ECU allowed independent activation/inactivation of the system, adjustment of ${PW}_{\mathrm{ref}}$ within safety limits, and recalibration of the pressure-to-stimulation mapping for each foot region. Functions were accessed via physical push buttons with on-screen prompts.

If sensations became uncomfortable and could not be corrected through recalibration, the participant was instructed to deactivate the system and return the external components for reprogramming. In such cases, study staff adjusted $W_{min}$, $PW_{max}$, and/or PA to restore comfortable, consistent, and safe percepts.

### Trial Design

Utilizing a quasi-experimental, time-series design, the study spanned 291 days (41 weeks) and was divided into three phases: Pre-Active SNP (29 days), Active SNP (219 days), and Post-Active SNP (43 days) (Figure 1F). Evaluations involving physical tasks and completion of written outcome measures were conducted at the beginning and end of each phase. Evaluations were also performed within the Active SNP and Post-Active SNP phases to further assess possible trends caused by continued home use and subsequent withdrawal of the system respectively.

The three trial phases were defined by the activation status of the SNP outside of the laboratory. The Active SNP phase was the only phase in which the user received sensory feedback during daily tasks at home and in the community. During the Pre-Active SNP phase and initial portion of the Post-Active SNP phase (prior to Post-Active 1), the participant was instructed to don the external system components, but it was inactive and not producing neural stimulation. Following Post-Active 1, all external SNP components were returned, and the participant resumed normal, daily life without SNP hardware or plantar sensory feedback.

To promote acclimation to the elicited percepts, the SNP recipient was instructed to perform structured weight-shifting and standing activities up to four times a week. To help mitigate changes in functional performance based on these exercises alone, the same activities were prescribed throughout the entire trial (from Pre-Active 1 to Post-Active 2). The exercise routine included forward and backward weight shifts to simulate phases of the gait cycle (heel strike to foot flat and foot flat to toe off), lateral weight shifts, and static standing in a modified tandem stance. These tasks were performed on firm ground and a compliant mat, with eyes open and closed. To foster sensory discrimination between the three established foot regions, the participant was instructed in a stair tap exercise where he would position his prosthetic foot on top of or against the lip of a step with his eyes closed and determine what portion of the foot was in contact with it. For all activities, he was permitted and encouraged to hold onto a sturdy object for support. A seated variation of these exercises was also provided to minimize upper extremity use and exacerbation of symptoms due to a shoulder injury sustained from a fall on ice unrelated to the study.

Prior to being sent home with the system, the SNP recipient demonstrated independence with all aspects of its operation and management including donning and doffing, securing cable connections, calibrating the pressure insole, charging the batteries, safely performing the sensory acclimation activities, and adjusting perceived stimulation intensities. Although the system would not be activated until the Active SNP phase, these tasks were practiced during earlier phases to ensure familiarity. Upon taking the system home, he was able to use the SNP as desired during normal, daily household and community activities despite active or inactive condition. On days the SNP was donned, a minimum wear time of four hours was recommended.

### Outcome Measures

#### Functional measures.

The following measures were assessed in the laboratory at each evaluation time point with the SNP donned but not delivering neural stimulation (inactive) and with the SNP actively providing neural stimulation (active). The order of SNP condition was randomized during each evaluation.

##### Timed Up & Go Test (TUG):

The TUG is a validated clinical test used to assess fall risk and balance [40,41]. The participant was instructed to rise from a seated position in an armed chair, walk 3 meters to a designated marker, turn around, return, and sit down. The task completion time was recorded for all trials. To assess whether elicited plantar sensory feedback influenced gait biomechanics, ground reaction forces (GRFs) were measured from three embedded force plates (AMTI OR6–6, Watertown, MA) at a sampling rate of 1,000 Hz. GRF data were low-pass filtered using a fourth-order Butterworth filter with a 10 Hz cutoff frequency. The SNP recipient was not instructed to modify gait or to specifically target the force plates. Only trials where a full step landed on force plates were included for analysis, with the assessment being repeated until a minimum of 5 full steps were obtained by each foot for each SNP condition. Each assessment time point used the same number of steps across conditions. The braking and propulsive force impulses were obtained by calculating the area under the anterior-posterior GRF curve from heel-strike to midstance and midstance to toe-off, respectively, during the stance phases of both the prosthetic and intact limbs.

##### Functional Gait Assessment (FGA):

The FGA is a 10-item clinical tool used to evaluate gait and postural stability across a range of walking tasks [42–45]. Each task is scored from 0–3, with a maximum score of 30. Higher scores indicate better functional performance. Tasks include level ground walking, gait speed changes, walking with horizontal and vertical head turns, pivot turns, obstacle negotiation, tandem walking, walking with eyes closed, walking backward, and stair negotiation. The FGA was performed twice at each evaluation, one per SNP condition, and scored by the same trained physical therapist throughout the study.

##### Stair Negotiation Task:

To examine the effect of plantar sensory feedback on stability and safety during complex terrain negotiation, a stair task involving varying step heights was developed. The participant ascended and descended stairs with 8” and 4” risers using a standard rehabilitation training staircase (tread depth = 9.5”). Each trial began by ascending one set of stairs, making a 90-degree turn at the top of the platform, and descending the alternate stairwell. Once both feet were placed at the base of the stairwell, he turned around and repeated the task in the opposite direction. Starting stair height would vary during each evaluation so that he would have initiated trials from both possible start positions. To limit reliance on visual feedback, commonly used by individuals with lower limb loss for balance [4], he carried a cafeteria tray to obstruct his lower visual field for half of the trials, so that an equal number of tray (obstructed view) and no-tray (clear view) trials were completed under each SNP condition. A minimum of 7 trials were performed per condition (e.g. SNP active/obstructed view), equating to at least 28 overall trials per evaluation. Completion times were recorded by a study staff member, and all trials were video recorded for follow-up analysis of negotiation strategy and errors (described below). Completion time, strategy, and error frequency were designated as outcome metrics.

Stair ascent and descent strategies were identified through video analysis and included changes in lead foot preference (prosthetic vs intact) and presence of heel probing, a compensatory strategy during descent where the user locates the next step by striking the posterior portion of the heel against the riser of the preceding stair. Stair errors were defined as an unintentional deviation from the instructed task or observable movement indicative of a loss of stability. Eight distinct error types were identified with three being exclusive to stair ascent, one exclusive to descent, and four indiscriminative of direction. Error types are listed and defined in Table 1.

Heel probing and error frequencies were calculated at each time point by dividing the cumulative observed events (heel probing or stair errors) by the total number of steps for each trial type (e.g. Active SNP with obstructed view during 4” stair ascent). This allowed for a direct comparison between the 4” and 8” staircases, as they had an unequal number of steps.

#### Self-Reported and Qualitative assessments.

Self-reported and qualitative assessments evaluated the participant’s experience with utilizing the SNP at home and in the community. Most assessments were evaluated at each time point, while some (i.e., usage diaries) were collected daily throughout the study.

##### Inventory of Falls and Stumbles:

At each evaluation, the SNP recipient reported the frequency of stumbles per week and number of falls since the previous assessment.

##### Questionnaires:

Validated questionnaires, including the Modified Prosthesis Evaluation Questionnaire (Modified PEQ), Falls Efficacy Scale–International (FES-I), Activities-Specific Balance Confidence Scale (ABC), and the 36-Item Short Form Health Survey (SF-36), were completed at each evaluation to assess factors such as prosthesis satisfaction, fear of falling, balance confidence, and quality of life.

##### Semi-structured Interviews:

To capture user experiences that may not be reflected in quantitative measures, a total of five semi-structured interviews were conducted across the trial: one within the Pre-Active phase (at Pre-Active 1), two within the Active phase (at Active 1 and 1 month prior to Active 2), and two during the Post-Active phase (at Post-Active 1 and Post-Active 2). All interviews were conducted by trained study staff, audio recorded, and transcribed verbatim. The resulting text was imported into NVivo 12 Plus (QSR International, Australia), and qualitative analysis was performed based on a modified grounded theory approach [46]. Detailed methodology has been previously described in Schmitt et al., 2023 [47].

##### Daily Diaries:

A structured diary was completed at home to document SNP wear time and notable experiences. The SNP recipient was provided duplicative printed handouts containing the same daily questions for each corresponding phase of the trial. After completion of each day’s diary, he was instructed to seal the entry within a manilla envelope and to not open, review, or edit it. Entries were returned to the lab at each evaluation time point except for Pre-Active 1 as this marked the commencement of the trial and diary usage.

### Statistical Analyses

For stance time and ground reaction forces measured during the TUG test, we performed a two-way repeated-measures ANOVA to determine the effect of SNP condition (i.e., inactive vs. active) over time. The normality of data distributions was determined using Shapiro-Wilk’s test. If there were any statistically significant interactions between the stimulation mode and time, we analyzed simple main effects. If a significant main effect was observed, post-hoc comparisons were performed using a Bonferroni correction to adjust for multiple comparisons (α =0.05).

For categorical data collected during the stair negotiation task, lead foot preference during ascent, counts of heel probing during descent, and discrete number of errors, were reduced to counts of “event” and “no event” at each evaluation for both active SNP and inactive SNP conditions. Given that each trial consisted of only 6 and 3 steps, respectively, for the 4” and 8” staircases, aggregating over the total number of steps for that condition provided stable denominators. These two-category counts were analyzed with a binomial generalized linear model (logit link) that included fixed effects for evaluation, SNP condition, and their interaction. Omnibus effects were assessed with likelihood-ratio chi-square tests. When the evaluation effect reached significance, pairwise contrasts of estimated marginal means were conducted. To control for type I errors inherent in multiple comparisons, the Holm-Bonferroni correction was applied. All statistics describe within-participant change only; no inference is made beyond this single case.

Statistical analyses were performed using either IBM SPSS Statistics Ver.22 (Chicago, IL) or R version 2022.02.0 (Austria).

## Results

### Extended SNP Use Increases Propulsive Force and Limb Propulsion Symmetry During Timed Up and Go

**Figure 2.**
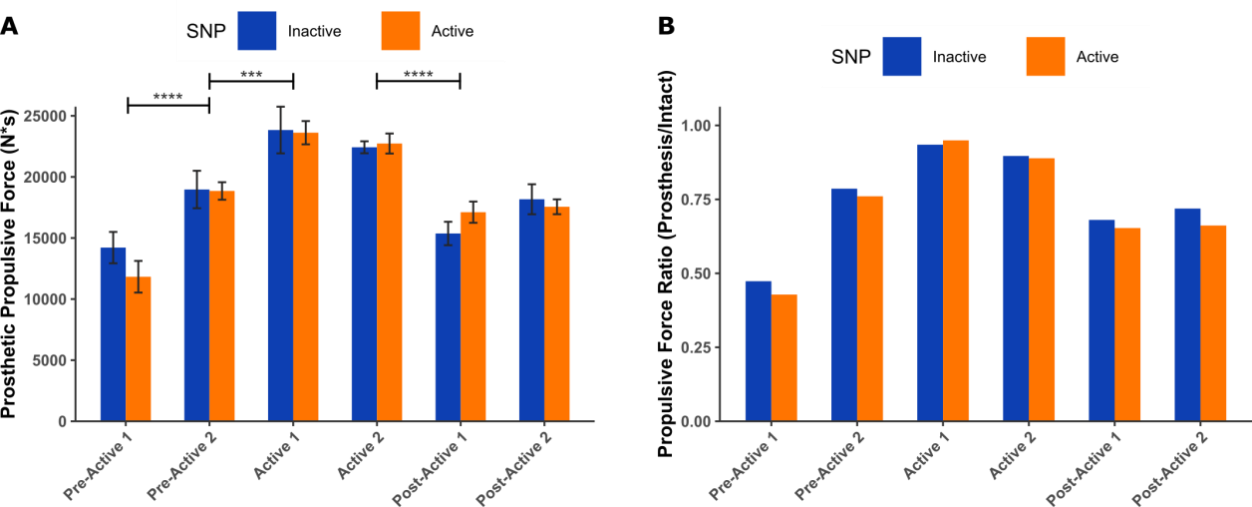
Propulsive force metrics during the Timed Up and Go (TUG) test across trial phases. (A) Propulsive force impulse of the prosthetic leg. (B) Ratio of the means between prosthetic to intact limb propulsive force impulses, indicating changes in limb propulsion symmetry over time. *** and **** indicate p < 0.001 and p < 0.0001

The propulsive force impulse of the prosthetic leg significantly changed across assessment time points (*F*(5,60)=25.04, *p*<.001, η^2^=0.68), with higher values observed during the Active SNP phase compared to both Pre-Active and Post-Active phases (Figure 2A). Similarly, limb propulsion symmetry, measured as the ratio of the prosthetic to intact leg propulsive force, improved during the Active phase (Active 1: 95% for active SNP; 94% for inactive SNP; Active 2: 89%, 90%) compared to Pre-Active 1 (43%, 47%), Pre-Active 2 (76%, 78%), Post-Active 1 (65%, 68%), and Post-Active 2 (66%, 72%) (Figure 2B). No significant overall effects were detected between active and inactive SNP conditions across all evaluations. Other outcome measures, including TUG completion time and stance time, did not show statistically significant differences across time points.

### SNP Activation Influences Functional Gait Assessment Scores

The highest FGA scores were observed during SNP activated conditions at Pre-Active 1 and Active 1, achieving 28 out of 30 points (Table 2). At these time points, the difference between active and inactive SNP scores met the minimum clinically important difference (MCID) threshold of 4 points. Because no MCID has been established for individuals with lower limb loss, the MCID value was derived from community-dwelling older adults as it most closely fit the demographic of our participant [44]. Across all evaluations, score differences between SNP conditions varied slightly. In general, performance was better on the second trial regardless of SNP condition, suggesting a potential practice effect. Exceptions were noted at Active 2, where performance was better in the first condition (SNP active) by 3 points, and at Post-Active 1, where identical scores were recorded.

### SNP Induces Shifts in Self-Selected Stair Negotiation Strategies and Error Frequency

**Figure 3.**
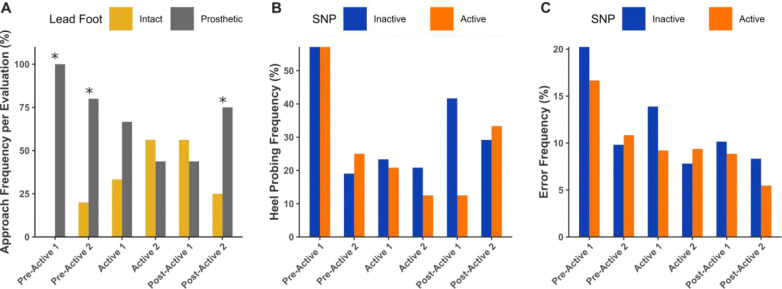
Changes in stair negotiation strategy and errors during stair navigation. Trials were performed on 4” stairs with an obstructed view and assessed across trial phases and SNP conditions. (A) Percentage of trials initiated with the prosthetic or intact limb at each evaluation phase. Asterisks mark phases whose prosthetic-versus-intact distribution differs significantly from the distribution pooled across all other phases. (B) Heel probing frequency during descent, compared between SNP conditions (Active vs. Inactive). (C) Error frequency during ascent and descent combined, also compared between SNP conditions. Counts are pooled across all trials for this participant; therefore, variability bars are not displayed.

Lead foot choice during 4” stair ascent varied across the study phases, but the global Pearson χ^2^ test did not reach the 0.05 threshold (χ^2^ = 10.0, df = 5, p = 0.074). Exploratory Holm-adjusted pairwise comparisons, however, showed that the distribution at Pre-Active 1 (p < 0.001), Pre-Active 2 (p = 0.009), and Post-Active 2 (p = 0.009) differed from the pooled remaining phases (Figure 3A). At Pre-Active 1, he consistently led with the prosthetic limb (100%), with a slight reduction at Pre-Active 2 (80%). During the Active phase, however, this preference diminished significantly as he chose to lead with his intact leg in 33% and 56% of trials at Active 1 and Active 2, respectively. This shift persisted into Post-Active 1 (56% intact lead). However, as time progressed during the Post-Active phase, his preference appeared to revert to the prosthetic limb, reducing selection of the intact foot to only 25% of trials at Post-Active 2. These results suggest a transient shift toward intact limb initiation during and immediately after the Active phase, although the overall change across time points did not achieve conventional significance.

Heel probing by the prosthetic foot during 4” stair descent appeared to occur more frequently with an inactive SNP during the Active and early Post-Active phases of the trial (Figure 3B). At Pre-Active 1, heel probing was performed equally between SNP conditions (57%). By Pre-Active 2, utilization of this strategy declined (25% active, 19% inactive) and remained low during Active 1 (21% active, 23% inactive). By Active 2, heel probing was more frequent in the inactive condition (21%) compared to active (13%). This difference increased at Post-Active 1 (42% inactive vs. 13% active), representing the largest disparity in heel probing frequency observed throughout the trial. By Post-Active 2, the incidence of heel probing for both active (33%) and inactive (29%) SNP conditions returned to values similar to one another and to those observed at Pre-Active 2. The binomial logit model confirmed a main effect of evaluation (likelihood-ratio χ^2^ = 16.5, df = 5, p = 0.0055), indicating that heel probing odds changed over time. SNP activation showed no independent effect (χ^2^ = 0.80, df = 1, p = 0.37) and did not interact with time (χ^2^ = 4.65, df = 5, p = 0.46). Holm-adjusted pairwise contrasts did not reveal significant differences between specific evaluation points.

The highest error rates (Figure 3C) occurred at Pre-Active 1 (20% inactive, 17% active), likely due to initial unfamiliarity with the task. Error frequency declined by Pre-Active 2 (10%, 11%) and remained stable during Active and Post-Active phases. Aside from Pre-Active 2, errors were higher with an inactive SNP, with the largest difference observed at Active 1 (14% inactive vs. 9% active). By Post-Active 2, error frequency decreased to its lowest levels (8% inactive, 5% active). A binomial logit model confirmed a significant main effect of evaluation on the odds of committing an error (likelihood-ratio χ^2^ = 63.23, df = 5, p < 0.001). Odds at every subsequent evaluation were three- to six-fold lower than at Pre-Active 1, with the lowest estimated probabilities during Active 2. SNP activation produced no independent effect (χ^2^ = 1.84, df = 1, p = 0.18) and did not interact with evaluation (χ^2^ = 7.50, df = 5, p = 0.19); pairwise contrasts between device states at each evaluation were not significant after Holm adjustment.

No meaningful trends or condition-specific effects were observed during tasks involving 8” stairs or clear view trials. Therefore, detailed analysis focused on 4” stair negotiation under obstructed view conditions, where notable changes in negotiation strategies and performance were identified.

### Stumble Frequency Declines with Home Use of a SNP

During the trial, the participant fell at home one time due to slipping on ice. This event occurred early in the Active phase, several days after completing the Pre-Active 2 evaluation. The incident was not related to the SNP, as the system was not donned at the time, but was reported for completeness and to adjust subsequent expectations and experimental procedures accordingly.

**Figure 4.**
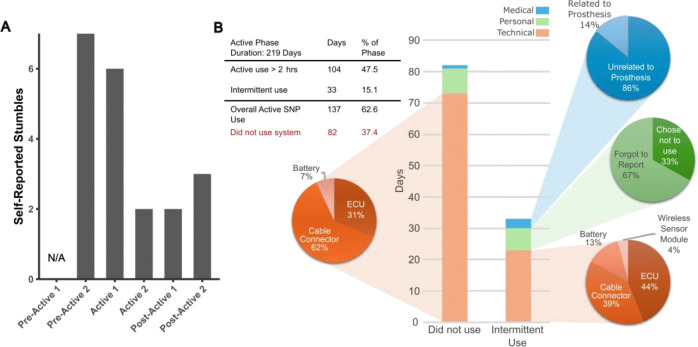
Weekly stumble frequency and SNP analysis of usage during the Active phase of the trial. (A) Self-reported frequency of stumbles at each evaluation, based on preceding week. (B) Daily SNP usage with a total of 137 days of activity (62.6% of the Active phase), including full and intermittent use. Reasons for non- or intermittent use were categorized as technical, personal, or medical. The medical reason for nonuse (accounting for 1 day of the trial) was due to experiencing unpleasant phantom sensory phenomenon, while personal reasons for nonuse were entirely (100%) due to choice, simply deciding not to don the system that day.

Weekly stumble frequency, as shown in Figure 4A, had the highest reported incidence at Pre-Active 2, with an average of 7 stumbles per week. Frequency declined slightly to 6 stumbles per week by Active 1 and then markedly dropped to 2 per week by Active 2. This lower rate was maintained through Post-Active 1 before increasing to 3 stumbles per week at Post-Active 2.

Results from other written outcome measures are reported in supplementary materials (Supplementary Figures 1–4).

### Usage Analysis Uncovers Barriers to Daily Use

The SNP was employed 137 out of 219 days of the Active phase, corresponding to a 62.6% usage rate (Figure 4B). This demonstrates consistent integration of the system into daily life over the 9.5-month period. To better understand usage variability, wear time records documented in daily diaries and participant communications were reviewed and categorized based on observed patterns. Days of limited or missed use were grouped as either “Did Not Use” (not worn, or lack of access to the system) or “Intermittent Use” (<2 hours). Contributing factors were classified as technical, medical, or personal as defined below.

Technical issues were the main contributor to intermittent and lack of use and primarily involved cable/connector faults and ECU software malfunction. If a technical issue arose, the external system components often had to be sent back to the laboratory for inspection or repair. This led to longer periods in which the system could not be utilized due to shipping and repair times.

Personal and medical reasons had a minor impact on overall SNP usage patterns. Personal reasons contributed to 10% of nonuse (8 days), and 9% of intermittent use (3 days), while medical reasons accounted for 1% of nonuse (1 day) and 21% of intermittent use (7 days). Notably, 86% of the medical disruptions resulting in intermittent use were unrelated to the prosthesis and mostly involved shoulder pain and occasional illness, while prosthetic-related intermittent use was due to receiving a new socket with a temporary foot and choosing not to utilize the SNP with these temporary pieces. Personal reasons for intermittent use were either due to the SNP recipient choosing not to utilize the system (33%) for more than 2 hours that day, or him forgetting to monitor and document usage (67%). Personal reasons for nonuse were entirely (100%) due to choice, with the participant opting not to don the system those days, while the presence of unpleasant phantom sensory phenomenon was the sole medical reason for nonuse.

### Changes in Charge Delivered after Extended Use

**Figure 5.**
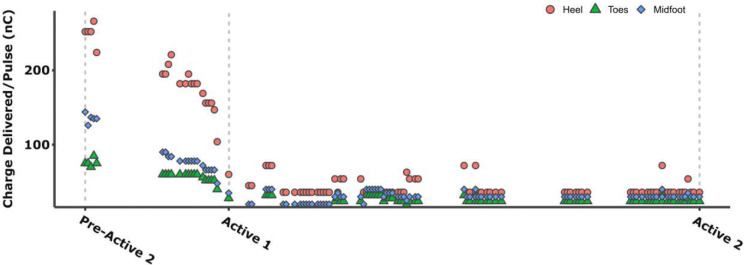
Changes in self-selected perceived stimulation intensity (charge delivered). Data reflects changes across foot regions (Toes, Midfoot, Heel) throughout the Active phase of the trial (Pre-Active 2 to Active 2) as reported in daily diaries.

During the Active phase, the SNP recipient independently adjusted the intensity of the perceived sensations for each foot region. He initially selected higher levels, which were later reported to make elicited sensations difficult to utilize. He subsequently reduced the intensities, leading to significantly lower delivered charge between Active 1 and Active 2 compared to the earlier portion of the Active phase (Figure 5). From Pre-Active 2 to Active 1, he used markedly higher charge for the heel region compared to those for the toe and midfoot. In contrast, the self-selected intensities were more consistent across regions from Active 1 to Active 2, with the heel still receiving the highest intensity, followed by the midfoot and toe.

### Qualitative Analysis of Interviews and Questionnaires

Semi-structured interviews conducted throughout the trial provided detailed descriptions of the user’s experience, which provided additional insight to contextualize the quantitative measurements. The sections below are two of twelve primary nodes that emerged from the qualitative analysis and are most relevant to the quantitative data presented here. Each node includes exemplary quotations from the participant to illustrate the main concepts. A full description of the qualitative analysis process, codebook, and resulting theoretical model were reported in a prior manuscript [47].

### Stimulation Level

User adjustments to the PW_ref_ affected the quality and intensity of evoked percepts. Remarks about percept changes were captured in the “Stimulation Parameters” node. Throughout the study, the SNP recipient used terms such as ‘high’ and ‘low’ to describe differences in selected PW_ref_ values. Early in the Active phase, he set the PW_ref_ to a ‘high’ level, leading to a sensation he described as “harsh” and “revved up,” making it difficult to “feel through” and therefore utilize the stimulation functionally.

“…when I put them [the stimulation levels] up at the high level my foot gets very excited, maybe kind {of} even agitated.” (Interview 3)

Because ‘high’ perceived sensations were difficult to interpret, he reduced the PW_ref_ values, resulting in more usable stimulation intensities.

“Now that I am running them [the stimulation levels] down low, everything seems to be much calmer, comfortable, and I really believe, still effective.” (Interview 3)

At these lower levels, the elicited sensations were more intuitive and functionally relevant.

“[The sensory feedback] is pretty amazing. At the lower levels [of stimulation] that I started working with……it [the sensory feedback] feels very realistic.” (Interview 02)

Ultimately, changes in the intensity of the perceived sensation from high to low during the Active SNP phase enabled the participant to use the sensory feedback more seamlessly when performing daily activities.

### Changes to Strategies for Locomotion

Strategies for locomotion are the physical and mental mechanisms he described using when performing movements with the prosthesis. For example, the SNP recipient described adjusting his stance based on socket pressure through the residual limb and altering gait mechanics to maintain balance. At the commencement of the trial, he claimed to be confident in his walking ability and wasn’t sure how the SNP could offer improvement.

“I told them [the researchers] before I don’t know how much the research is going to help a guy like me because I do what I want to do, I walk just fine…. I’ll out-walk a lot of my friends. This [intact] knee will give me more problems than my prosthetic leg will.” (Interview 01)

However, he began noticing changes in locomotor strategies several months into the Active SNP phase of the trial, especially when visual feedback was limited. This realization became more evident when the SNP had been returned for repair and the participant was assisting a friend move furniture up a staircase. He described searching for plantar sensation by lightly tapping his prosthetic foot, then having to revert to his typical strategy of focusing on pressure through the socket of the prosthesis once neural stimulation was not available.

“…we were going up the steps and I couldn’t see my foot. I found myself standing, tapping the steps with my prosthetic foot trying to feel them… I realized that I was lacking my stim [neural stimulation] and I couldn’t find the step. And in order to find the step, I had to put a little more weight on it [the prosthesis] so I could feel it up to my stump and my leg and into my body.” (Interview 03)

Furthermore, he noted changes in weight shifting onto his prosthetic leg when he no longer had access to the SNP because the external components had been sent back to the lab for repair. Without plantar sensory feedback from the system, he described the need to shift more weight onto his prosthesis to receive sensation through interactions of his residual limb with the socket. In contrast, elicited plantar sensation from the SNP enabled him to determine prosthetic foot/floor interaction with less effort and applied pressure.

“[With] the stimulation I don’t have to touch the ground very hard to feel it [the stimulated sensation]. And when, when I don’t have stimulation (from the SNP) I have a little more weight on [the prosthesis] to get it to come up through my body. You know, I realized that my foot’s solidly on the ground.” (Interview 5)

## Discussion

With prolonged home SNP use, the system recipient demonstrated an increase in prosthetic limb propulsion and improved propulsive force symmetry between his prosthetic and intact legs. His self-selected strategies when negotiating an atypically short (4”) staircase also shifted, with the participant exhibiting a change in lead foot preference during ascent and decline in the use of compensatory gait strategies (heel probing) when visual feedback of foot position was restricted. These observed changes suggest the development of equal confidence in both limbs to effectively initiate ascent and reduced need to rely on altered gait mechanics to compensate for lack of visual feedback of limb position, thus promoting adoption of more normalized gait behaviors. Plantar sensory feedback also appeared to influence FGA performance and stumble incidence, with the participant achieving his highest FGA score solely when the SNP was activated and reporting a decline in weekly stumbles during the Active phase of the trial.

Whereas previous studies were restricted to laboratory use of the system, this study allowed for real world utilization of daily plantar sensory feedback over the course of multiple months. Observed functional improvements were noted primarily within the Active phase but were not exclusively dependent on SNP activation. This suggests that daily SNP use may enable broader changes in motor control strategies over time that persist during the short intervals between active SNP use, leading to overall improvements in gait biomechanics and functional performance. Once consistent daily SNP use is discontinued, however, these strategies could revert to previous performance standards.

Results from the TUG support this hypothesis, with the user exhibiting an increase in the propulsive force of his prosthetic limb (Figure 2A) and enhanced limb propulsion symmetry (Figure 2B) during the Active SNP phase. Once active home SNP use was discontinued, these metrics appeared to revert to values similar to those observed in the Pre-Active evaluations. Given that no significant disparity was noted between SNP conditions at each time point, these observations suggest that regular utilization of plantar sensory feedback can facilitate changes in motor control strategies that enhance utilization of the prosthesis and improve gait symmetry, which could potentially lessen the metabolic burden of the intact limb and help prevent associated overuse injuries such as osteoarthritis and low back pain [33,48]. Future studies should explore these long-term changes to enhance our understanding of motor adaptations following lower limb amputation and assist in the development of more effective rehabilitation protocols.

While clear changes in propulsive force were evident, the clinical impact on gait and postural stability was less conclusive. FGA scores fluctuated slightly throughout the trial, with his best score (28/30) achieved twice (Pre-Active 1 and Active 1), but only when the SNP was active (Table 2). These were the only assessments in which the MCID of 4 was obtained, suggesting that an active SNP can lead to clinically meaningful improvements in performance. However, these improvements must be interpreted cautiously, as the inactive SNP condition preceded the active condition at these two time points with only brief rest periods between assessments. Thus, a potential training effect cannot be eliminated for the condition that is performed second at each time point. Conversely, performing the inactive SNP condition second could result in utilization of transient motor adaptations that may have occurred during the initial testing with the active SNP. Despite these potential confounds, active SNP scores were greater at both Active phase evaluations despite performing the active condition first at Active 2. These results suggest that plantar sensory feedback has the potential to significantly improve dynamic postural stability, especially with daily use. Future studies would benefit from including a washout period of several hours or days between active and inactive conditions to provide a more robust evaluation of prolonged SNP exposure.

Clear changes in stair negotiation strategies were observed over the course of the interventional timeline. During Pre-Active evaluations, the SNP recipient demonstrated a strong preference to lead with his prosthetic foot when ascending the 4” stairs, which is common for individuals with unilateral transtibial amputations when navigating or crossing obstacles [7,49]. This prosthetic lead foot preference to initiate gait is arguably performed to combat the biomechanical shortcomings of the prosthesis which can limit propulsive force and impact the center of pressure trajectory if utilized as the trailing limb [50]. During the Active SNP phase, however, reliance on this strategy appeared to wane, with the participant initiating ascent with each leg almost an equal number of times by the Active 2 evaluation (Figure 3A). The sensory feedback provided by the SNP enhanced awareness of prosthetic foot positioning and contact, possibly improving prosthetic utilization by increasing the propulsive force during stair ascent. This improved stability likely increased confidence in supporting his weight entirely on the prosthetic limb while advancing his intact limb forward. Future work will expand the data collection to include propulsive forces during stair ascent and descent and postural sway to evaluate these force production and stability changes.

In addition to lead foot preference, changes in heel probing frequency were also observed across time points (Figure 3B), with the frequency being notably high during the first assessment. This observed high incidence at Pre-Active 1 likely reflects unfamiliarity with the task and limited experience navigating short stair heights without visual feedback. The reduction in heel probing observed at Pre-Active 2 could be attributed to a training effect, as Pre-Active 2 was conducted 3 weeks after Pre-Active 1. Although heel probing frequency continued to decline during the Active phase evaluations, the reduced incidence was not substantially different from that observed at Pre-Active 2. Unlike the Pre-Active evaluations, however, the possibility of a training effect during the Active phase assessments was unlikely due to the extended intervals between those evaluations, with lengths of 49 and 170 days respectively. This suggests that daily home SNP use can contribute to a reduction in compensatory behaviors such as heel probing.

During the Post-Active phase, the frequency of heel probing increased, suggesting that stair descent adaptations, like those observed in prosthetic propulsion force, tend to revert towards baseline conditions following withdrawal of daily SNP use. Although heel probing was employed almost equally between SNP conditions throughout the home use trial, a significant disparity was noted at Post-Active 1, where it was implemented markedly more often when the SNP was inactive. Given the short duration of only 2 weeks since the previous assessment, the absence of plantar sensory feedback at Post-Active 1 may have seemed more pronounced, causing him to reflexively revert to this compensatory strategy when the SNP was inactive. As these results strictly pertain to short stair heights, however, further experiments are necessary to see if the SNP has similar effects when ascending uneven terrain or slopes.

We also evaluated the frequency of errors committed during such stair negotiation tasks and hypothesized that SNP activation would reduce error frequency and improve completion time. Although having an active SNP typically resulted in a lower error frequency (Figure 3C), which could suggest that SNP use can lessen fall risk during stair negotiation, the observed differences between SNP condition and time points were not significant. Completion time also did not show much variability. Due to the high functioning nature of this SNP user, however, these results could reflect a potential ceiling effect. Error frequency and completion time may be more sensitive metrics for the impact of the SNP in individuals with greater physical and balance deficits.

There was a reported decline in weekly stumble frequency during the Active SNP phase which could be attributable to the observed gait and functional performance changes. Reduced stumble incidence could imply that daily SNP use can lessen fall risk by limiting the occurrence of this common precipitating event. As evident in Figure 4A, reports of stumbles declined during the Active phase of the trial and increased slightly during the Post-Active phase, though not to the extent seen in the Pre-Active phase. This reduction in stumbles reported at the end of the Active phase could be due to integration of normalized motor behaviors and better incorporation of the prosthetic foot into the body schema in response to frequent plantar sensory feedback. This is consistent with recent studies demonstrating improvements in multiple metrics of stumble recovery following short-term SNP use [51]. The slight increase in stumbles reported at Post-Active 2 could reflect a decline in the utilization of these learned behaviors and reduced positional awareness of the prosthesis due to withdrawal from daily SNP use. Research into fall risk prevention training in individuals with limb loss suggests that only a few weeks of training can lead to sustained benefits lasting multiple months [52]. Thus, prolonged SNP use alongside fall prevention training may enhance these improvements and induce long-term effects. Future home use trials would benefit from incorporating fall prevention training and adding follow-up examinations 3 to 6 months following the Active phase to determine if reductions in fall risk are maintained for longer periods after SNP use.

While there were instances when regular SNP usage was disrupted due to technical issues that required the external components to be returned for repair, it was during these abrupt discontinuations that the SNP recipient reported sudden awareness that he had unknowingly been incorporating the sensory information provided by the system into his motor planning and execution. For example, during the semi-structured interviews he described assisting a friend with carrying a table up a flight of stairs. Since the table prevented the compensatory use of visual feedback typically employed by individuals with lower limb loss, he instinctively searched for the next step by tapping the toes of his prosthetic foot, expecting to elicit the plantar pressure sensations generated by the SNP. When these sensations were not provided, he realized that he had subconsciously integrated the sensory feedback from the SNP into his actions, and that he relied on the SNP to facilitate various ambulatory and functional tasks [47]. This instinctive utilization of the prosthetic foot to perform searching tasks is uncharacteristic of individuals with unilateral limb loss, and attempting this strategy without elicited plantar sensation from the system may indicate that the behavior was learned over time throughout the Active SNP phase of the trial [34]. This further suggests that individuals with lower limb loss could better rely on their prostheses and possibly adopt more typical gait mechanics with consistent use of a SNP at home and in the community.

An additional aspect to consider during prolonged home use of the SNP is that selecting the appropriate stimulation levels could have a significant effect on both the quality and the sensorimotor integration of the evoked sensory percepts. During the semi-structured interviews, higher perceived stimulation intensities were associated with percepts that were more intense and therefore distracting, making them difficult to utilize functionally. Lower, yet still perceivable, stimulation intensities seemed to facilitate integration of the neural stimulation into functional tasks, such as searching for uneven ground, slopes, and stairs with the prosthesis [47]. These behaviors were even more evident when visual feedback of his prosthetic leg was blocked.

Due to the high functioning nature of the SNP recipient, clinically significant changes in balance confidence were not observed on standardized subjective measures like the ABC and FES-I (Supplementary Table 1 & 2), despite experiencing a slip and fall on ice unrelated to SNP use early in the Active phase of the trial. Although a small reduction in ABC score was observed at the evaluation following the fall (Active 1), the change was not clinically significant. A more generalized outcome measure with questions non-specific to lower extremity function and ailments, the SF-36 (Supplementary Figure 1), suggests that his responses may have been confounded by the social and emotional repercussions of the pandemic, particularly since he had sustained a shoulder injury that could not be surgically repaired until completion of the home use trial due to COVID-19 restrictions on elective surgeries. Therefore, more objective outcome measures that can specifically measure the influence of prosthetic use or lower extremity function should be utilized in future studies.

Finally, technical issues caused several disruptions to SNP use, and hardware improvements were identified and implemented to help mitigate these issues in future home use trials. These modifications include the development of a new, more energy efficient ECU, stronger attachments to secure internal electronic components of the wireless sensor module, increased durability of the cable connector, and development of an Android-based phone application to allow more intuitive activation and control of stimulation parameters.

Recommended modifications to future home use trials include increasing the trial length by extending the Active SNP and Post-Active SNP phases to provide more time to actively utilize the system, identify potential plateaus in functional improvement, and better identify trends following discontinuation of home use. To limit SNP use disruptions, technical issues could likely be mitigated through software upgrades and providing redundant components for immediate replacement. System recipients should also be introduced to outcome measures and assessments prior to the start of the trial to familiarize them with questions that will be asked and reduce the likelihood of substantial functional differences between the initial assessment time point and subsequent evaluations because of a training effect. Finally, electronic collection of daily diary information could be less time-consuming and more user friendly than the written records used in this study, and reducing this burden could improve adherence to the reporting regimen.

## Conclusions

Despite prosthetic advancements and utilization of compensatory gait and balance strategies, individuals with lower limb loss continue to exhibit increased fall rates compared to those living without limb amputation. This study extended and expanded our understanding of the functional impact of sensory neuroprostheses beyond previously reported improvements in postural stability, performance of an ambulatory searching task, gait symmetry, and stumble recovery to encompass the effects of prolonged home use on function, balance confidence, quality of life, and fall and stumble frequency. The results of this initial extended trial indicate that prolonged home use of a sensory neuroprosthesis can increase propulsive force symmetry between the prosthetic and intact limb, promote adoption of more typical gait behaviors, decrease stumble frequency, and provide users with meaningful plantar sensations that can be utilized similarly to those from the intact limb when performing tasks of daily living at home and in the community. Collectively, these positive effects may eventually contribute to safer mobility, better long-term musculoskeletal health, and a more confident, engaged personal and social life. Further studies are required to better capture specific changes to gait biomechanics due to electrically elicited plantar sensory feedback, assess prosthetic embodiment, and determine the replicability of these results to others living with lower limb loss.

## Supplementary Material

Supplementary Files

This is a list of supplementary les associated with this preprint. Click to download.

• Homegoing2020SupplementaryTablesandFigures.docx

## Figures and Tables

**Table 1.** Stair error types with definition

Stair Direction	Error Type	Definition
**Stair Ascent**	Toe Hits Front Step	When attempting to place foot on step, the foot is advanced too far forward and the front/toe portion of shoe kicks the riser of the next step
	Toe Slides Along Edge of Step	When attempting to advance foot from one step to the next, the front/toe portion of the shoe scrapes along stair nosing
	Full Knee Extension with Subsequent Instability	When attempting to place foot on step, foot is positioned with heel hanging too far off edge of stair, causing sudden knee extension when body weight is shifted onto limb
**Stair Descent**	Heel/Side of Foot Slides Along Edge of Step	When attempting to lower foot onto next step, the foot is not advanced far enough forward, and the heel/side of the foot remains or slides along the edge of the preceding stair
**Bidirectional**	Touches/Grab Handrail	Participant suddenly reaches for and touches/grabs handrail
	Leg Hits Other Leg	Prosthetic foot kicks or is kicked by intact foot
	Restarts Attempt	Participant advances foot forward but then returns foot to the start position or preceding step before attempting advancement again
	Skips Step	Foot advances too far forward and step is skipped

**Table 2.** Influence of plantar sensation on Functional Gait Assessment (FGA) performance.

	Pre-Active 1*	Pre-Active 2	Active 1*	Active 2	Post-Active 1	Post-Active 2
**SNP Inactive**	**24**	26	**24**	23	23	25
**SNP Active**	28	**23**	28	**26**	**23**	**24**

Scoring ranges from 0–30 with higher values indicating better performance. Bolded numbers denote initial condition performed. Asterisks (*) indicate an improvement at or higher than the MCID between SNP conditions.

## Data Availability

The data that support the findings of this study are available upon reasonable request from the corresponding author, H.C.

## List of Abbreviations

SNP: Sensory Neuroprosthesis
ECU: External Controller Unit
C-FINE: Composite Flat Interface Nerve Cuff Electrode
PW: Pulse Width
PA: Pulse Amplitude
IPI: Interpulse Interval
TUG: Timed Up-And-Go Test
GRF: Ground Reaction Force
FGA: Functional Gait Assessment
PEQ: Prosthesis Evaluation Questionnaire
FES-I: Falls Efficacy Scale–International
ABC: Activities-Specific Balance Confidence Scale
SF-36: 36-Item Short Form Health Survey
MCID: Minimum Clinically Important Difference
